# Effects of night-to-night variations in objectively measured sleep on blood glucose in healthy university students

**DOI:** 10.1093/sleep/zsae224

**Published:** 2024-09-26

**Authors:** Alyssa S C Ng, E Shyong Tai, Michael W L Chee

**Affiliations:** Sleep and Cognition Laboratory, Centre for Sleep and Cognition, Yong Loo Lin School of Medicine, National University of Singapore, Singapore, Singapore; Department of Medicine, Yong Loo Lin School of Medicine, National University of Singapore, Singapore, Singapore; Sleep and Cognition Laboratory, Centre for Sleep and Cognition, Yong Loo Lin School of Medicine, National University of Singapore, Singapore, Singapore

**Keywords:** sleep duration, naps, wearable sleep tracking, CGM, blood glucose, insulin resistance, university students

## Abstract

**Study Objectives:**

We examined associations between daily variations in objectively measured sleep and blood glucose in a sample of non-diabetic young adults to complement laboratory studies on how sleep affects blood glucose levels.

**Methods:**

One hundred and nineteen university students underwent sleep measurement using an Oura Ring 2 and continuous glucose monitoring (CGM) for up to 14 days. In 69 individuals who consumed a standardized diet across the study, multilevel models examined associations between sleep duration, timing, efficiency, and daily CGM profiles. Separately, in 58 individuals, multilevel models were used to evaluate postprandial glycaemic responses to a test meal challenge on 7 days. Participants also underwent oral glucose tolerance testing once after a night of ad libitum sleep, and again following a night of sleep restriction by 1–2 hours relative to that individual’s habitual sleep duration. Between-condition glucose and insulin excursions, HOMA-IR and Matsuda index were compared.

**Results:**

Nocturnal sleep did not significantly influence following-day CGM profiles, postprandial glucose, or nocturnal mean glucose levels (all *p*s > .05). Longer sleep durations were associated with lower same-night glucose variability (all *p*s < .001). However, the range of variation in sugar levels was small and unlikely to be of functional significance. Considering naps in the analysis did not alter the findings. Sleep restriction by an average of 1.73 hours (*SD* = 0.97) did not significantly impact excursions in glucose or insulin or insulin sensitivity the following morning (all *p*s > .05).

**Conclusions:**

Glucose handling in young, healthy adults may be more resilient to real-life fluctuations in sleep patterns than previously thought.

**Clinical Trial Information:**

Monitoring Sleep and Glucose Among University Students https://clinicaltrials.gov/study/NCT04880629, ID: NCT04880629

## Graphical Abstract

**Graphical Abstract:**
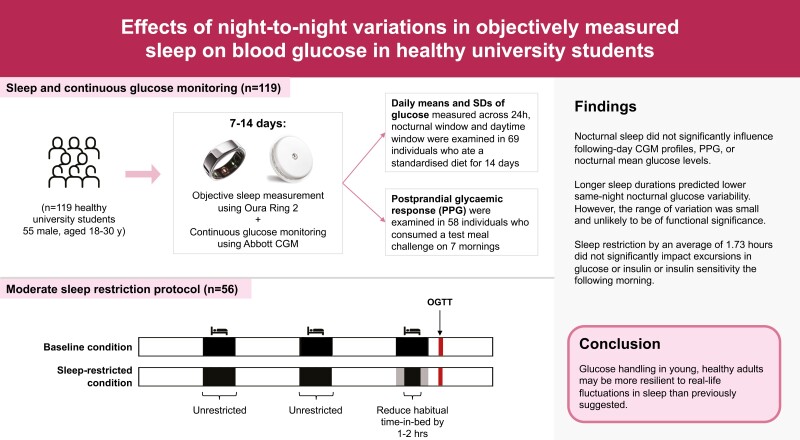

**Statement of Significance**

Many experimental studies suggest that sleep loss is linked to impaired glucose metabolism and higher diabetes risk. However, whether nightly variations in sleep affect glucose metabolic outcomes in free-living, non-diabetic populations has not been shown. In healthy college students, daytime glucose profiles were not affected by the previous night’s sleep. Insulin levels and insulin sensitivity were also not affected by moderate sleep restriction, suggesting that glucose handling in young, healthy adults may be more resilient to real-life fluctuations in sleep patterns than previously thought. These findings also contrast with earlier work in the same sample that showed fluctuations in sleep were tightly associated with next-day well-being. There thus appear to be differential effects of sleep restriction on affective and glucose metabolic outcomes.

A large body of experimental and epidemiologic evidence supports a strong association between short sleep duration or poor sleep quality and a higher risk of developing diabetes, obesity, and cardiometabolic disorders [1–6]. Experimental studies have shown that short-term restriction of sleep to 4–5 hours per night for up to a week can result in impaired glucose tolerance, lowered insulin sensitivity, and elevated blood glucose levels [7–9]. Data from cross-sectional and prospective epidemiological studies reported associations between short sleep (<6 hours/night) and higher incidence of metabolic syndrome or diabetes mellitus [10–12]. In persons with diabetes, the amount and quality of sleep have also been found to influence glucose handling [13–15]. Collectively, these data strongly suggest that short or shortened sleep may adversely affect glucose metabolism and elevate the risk of diabetes mellitus.

However, in practice, the effect of naturalistic night-to-night variations in real-world sleep on blood glucose levels has not been adequately characterized. Much of our current understanding of the relationship between sleep and blood glucose is based on evidence from experimental studies that compared the effects of sleep restriction of 5 hours or less per night on multiple successive nights relative to control schedules where 8–10 hours of sleep were provisioned [7–9]. In practice, sustained versions of these “long” and “short” sleep are uncommon in real life. Instead, in many students and working adults, one or two nights of short sleep are often followed by nights of slightly longer sleep duration. More moderate variations in sleep are driven by fluctuating school, social, or work demands on weekdays, and “catch-up” sleep on weekends or free days [16–18]. Thus, it is unclear whether the results of lab-based studies on short sleep generalize to the real world.

Objective, longitudinal measurement of at-home sleep and blood glucose levels could help clarify how variations in sleep timing and duration affect blood sugar in an ecologically relevant manner. The advent of wearable continuous glucose monitoring (CGM) systems, that can passively monitor blood glucose levels over long periods of time, has made this endeavor eminently feasible [19]. Several studies have explored how natural sleep affects CGM-derived glucose profiles among individuals with type 1 and 2 diabetes [20–23]. However, despite the growing use of CGM by health enthusiasts [24, 25], only one study to date has examined sleep duration-blood sugar associations in healthy adults [26]. Furthermore, as the insulin response to rising glucose levels differs between diabetic and non-diabetic individuals [27, 28], it is also uncertain how fluctuations of nocturnal sleep duration affect blood glucose in healthy persons.

The present work examined the effects of nightly variations in sleep on blood sugar profiles measured continuously with CGM for 2 weeks in non-diabetic college students living in situ. In the first of two related studies, we examined associations between sleep and glucose levels measured across the whole day, as well as over a daytime and nocturnal window. We catered macronutrient-calibrated meals to attenuate potentially confounding effects of changes in sleep on composition, quantity, or quality of food intake, and downstream from these, on glucose levels [29–33]. This was to ascertain whether less healthy sleep habits like sleeping shorter, later, or more variably by themselves could adversely affect blood glucose levels. We hypothesized that nights with shorter sleep duration, lower sleep efficiency (SE) and later sleep timing would be associated with higher, more variable blood glucose profiles.

Preliminary analyses of this data suggested that there were no significant effects of sleep on glucose profiles measured during the day. This prompted us to add the second study, in which we assessed associations between sleep and postprandial glycemic responses to a standardized liquid breakfast repeated on 7 days. Other than the standardized breakfast, participants were allowed to eat ad libitum. This allowed us to test whether previous negative findings in Study 1 were a result of limiting nighttime food intake, which ordinarily might increase on nights with shorter sleep. We additionally examined whether a night of moderate (1–2 hours) added sleep restriction over that person’s habitual sleep schedule would affect insulin sensitivity, glycemic response, and insulin response to a glucose load. Based on previous findings, we expected that this added moderate sleep restriction would decrease insulin sensitivity and increase postprandial glucose and insulin excursions.

## Methods

### Participants and inclusion criteria

Ethics approvals for all procedures were obtained from the Institutional Review Board of the National University of Singapore. Participants were briefed and provided written consent prior to participating in any research procedures. They received financial compensation for taking part in this research.

**Figure 1.**
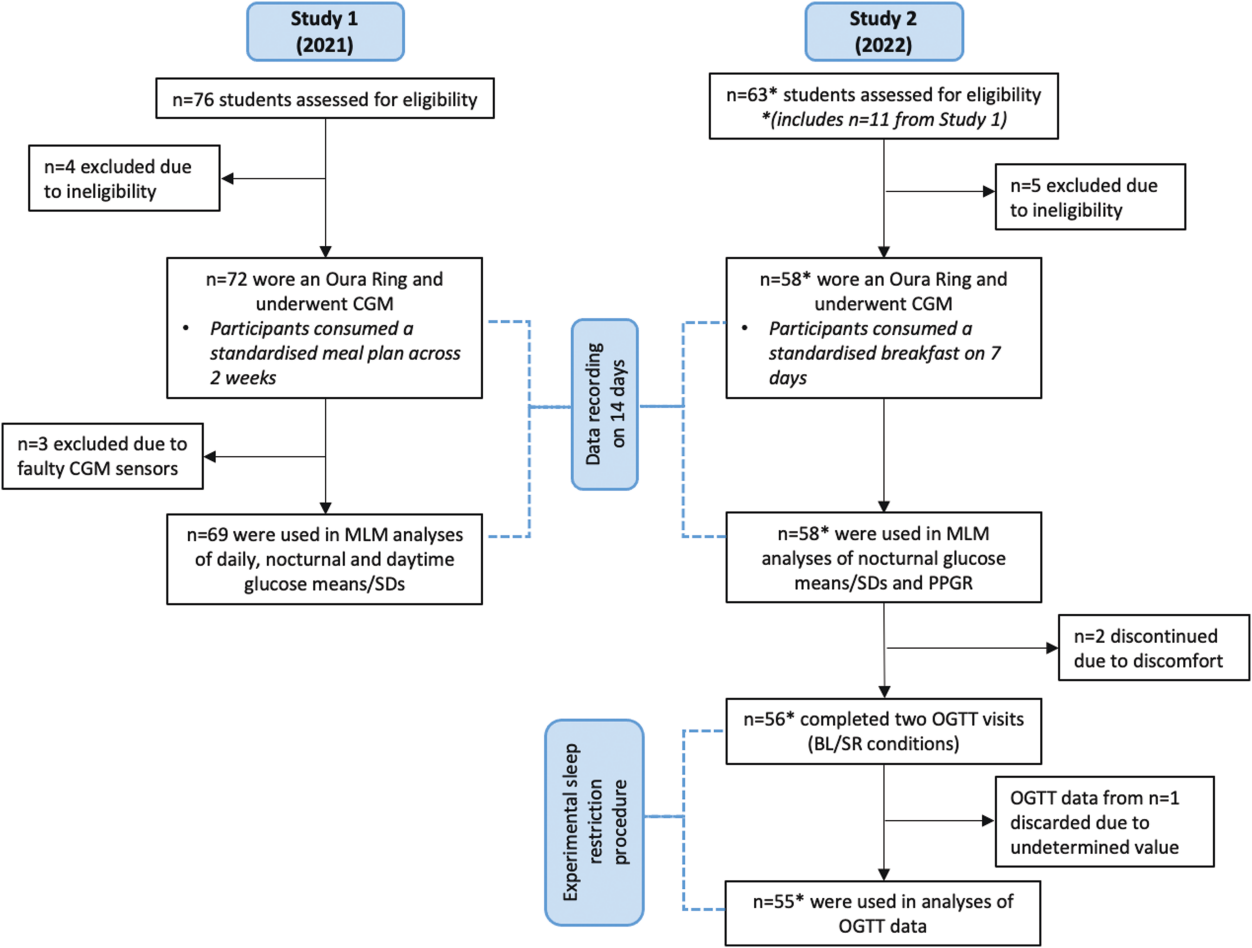
Flowchart showing key features of study 1 and 2 including participant numbers and procedures

Data were collected from a total of 119 participants (55 male; mean age 22.54 ± 1.74) from two related studies (study 1 [2021]: *n* = 72 [33 male; mean age 22.16 ± 1.74]; study 2 [2022]: *n* = 47 [22 male; mean age 23.12 ± 1.59]). Eleven participants in study 1 also took part in study 2, hence a total of 58 participants took part in study 2. A recruitment flowchart is shown in Figure 1.

Participants were students at the National University of Singapore, recruited via advertisements posted on the university’s learning platform or hostel mailing list. Qualifying participants had no known history of diabetes mellitus, systemic disease, neurological, psychiatric, or sleep disorders. Individuals with a BMI > 25.0 kg/m^2^, or with moderate to severe risk of sleep-disordered breathing assessed using the Berlin Questionnaire, moderate to severe risk of anxiety (Beck’s Anxiety Inventory category score ≥3) or moderate to severe risk of depression (Beck’s Depression Inventory score ≥3) were also excluded.

Participants in study 1 had to be living on campus during the study to facilitate the distribution of catered meals at specific time windows. Study 2 had no restrictions on whether participants were hostel dwellers or living off campus. Participants in study 1 additionally underwent screening for impaired glucose tolerance via a 2-hour oral glucose tolerance test (OGTT) during a baseline laboratory visit. Three individuals indicating a glycated hemoglobin (HbA1c) level of ≥6.5% or abnormal 2-hour OGTT plasma glucose level (>7.8 mmol/L) were identified for exclusion using these criteria. However, after a repeat assessment 2 weeks later, all three were found to have normal OGTT and HbA1c results and were allowed to continue their participation in the study.

### Data collection procedure (14 days)

In both studies, participants attended a baseline laboratory visit, during which they were briefed on study procedures and completed a set of demographic and lifestyle questionnaires. Eligible participants were fitted with a wearable sleep and activity tracker (Oura Ring, Oura, Health Oy, Oulu, Finland) and downloaded apps used for data collection onto their smartphones via the Google Play store (Oura client app, Z4IP Ecological Momentary Assessment app, FoodView food diary application). Research staff then trained participants to use the Oura Ring and smartphone apps for data recording during the following 14 days. CGM sensors were applied by trained research staff on the morning of the first day of the study.

### Sleep assessment

In both studies, sleep information was recorded using a validated consumer wearable sleep and activity tracker (Oura Ring 2, Oura, Health Oy, Oulu, Finland). Previous evaluations of the Oura Ring’s sleep–wake detection accuracy in our laboratory and by others have established the device to perform well when tested with polysomnography and research-grade actigraphs [34–38]. Its performance holds up well against other consumer wearables in standard laboratory testing across young and old adults [39], as well as under a novel protocol designed to confound accurate sleep–wake classification through the insertion of periods of relatively motionless wakefulness [40].

Participants were instructed to always wear the ring on their non-dominant hand, except when the device was being charged or when playing contact sports. Sleep–wake periods were estimated using Oura’s proprietary algorithm which takes into account body movement, heart rate variability, a circadian factor, and temperature [41]. Nightly bedtime, wake-up time, time-in-bed (TIB), total sleep time (TST), sleep onset latency, and wake after sleep onset were measured. The proprietary sleep assessment algorithm was locked and was thus identical for all participants throughout the studies. SE was calculated as 100 × (TST/TIB), and mid-sleep time (MST) was calculated as the midpoint between bedtimes and wake-up times as assessed by the device. Variability in a participant’s sleep duration was determined thus: standard deviations (SD) of TST were calculated for each week of measurement (containing sleep episodes on a minimum of 4 weekdays and 1 weekend day), and the average was taken across all qualifying weeks.

Self-reported bedtimes and wake times were recorded on an electronic diary (Z4IP Ecological Momentary Assessment app) and used only to check sleep period times recorded by the Oura Ring. Nap occurrence and duration in minutes were recorded on the app each evening, in response to the question “How long did you nap for today?.” Participants could record their responses on a sliding scale (0–120 minutes). Participants in both studies also used this app to record ratings of their subjective well-being twice daily (for 4 weeks in study 1; 2 weeks in study 2); findings of this evaluation have been published elsewhere [42].

### Continuous glucose measurement

Participants in both studies wore a wearable CGM device on their upper non-dominant arm for a consecutive 14-day period. Participants in study 1 (*n* = 72) wore the FreeStyle Libre Pro (Abbott, Abbott Park, IL, USA), while participants in study 2 (*n* = 58, inclusive of 11 participants who also took part in study 1) wore the newer model (FreeStyle Libre Pro iQ), due to the discontinuation of the FreeStyle Libre Pro in between studies. Both devices store glucose values in 15-minute intervals for up to 14 days. Participants could not view their glucose values during the recording period. On the morning of the first day of recording, the CGM sensor was attached to the participant’s upper, non-dominant arm by trained research staff. The sensor was subsequently scanned at regular intervals during the study by research staff (at least once per week) to ensure it was still functioning properly. A sensor requiring reattachment was replaced with consent from the participant for the remainder of the 14-day period.

Following advice from Abbott, data collected on the first day for each CGM were removed to avoid inaccuracies caused by calibration during the first 12-hour window after application.

### Standardized whole-day food intake (study 1)

Seventy-two individuals participating in study 1 consumed a standardized meal plan across the 14-day study. On each day, breakfast, lunch, dinner, and snacks were provided to participants, with instructions to refrain from consuming other foods. Participants were allowed to consume caffeinated beverages (with no added sugar). Meals were designed by a dietician and prepared by caterers to match the macronutrient composition of an average Singaporean diet (50%–60% carbohydrates, 15%–20% protein, 30%–40% fat; taken from the National Nutritional Survey, 2018). Total calories per day were approximated at 2200 kcals for male participants and 1800 kcals for female participants per the Ministry of Health recommendations for young adults of average weight based on the Oxford equation [43]. Four whole-day menus consisting of breakfast, lunch, dinner, and snacks were rotated across the 14 days such that each was repeated on multiple days. Menus were not systematically different in macronutrient composition. Participants collected the meals at their hostel within fixed windows (breakfast: 0730–0900 hours; lunch: 1130–1300 hours; dinner: 1800–1930 hours) and were instructed to finish meals within 2 hours of collection.

To ensure adherence to this meal plan, participants were instructed to submit to research staff before and after photos of all food and caloric beverage intake documented with a food photo diary application installed on their smartphones (FoodView app). Photos were visually rated by research staff to determine whether meals were finished (“YES” if ≥75% finished, no components left uneaten, no external foods pictured), with discussion where uncertain. (See example meal photos in Supplementary Figure 1).

### Assessment of postprandial glycemic response (study 2)

Participants in study 2 consumed a test meal for breakfast consisting of a chocolate-flavored packaged drink (Ensure Plus Milkshake Chocolate, 200 mL, Abbott) on seven consecutive days during the 14-day recording period. They were instructed to finish the drink within 10 minutes, not to eat or drink any other foods or beverages prior to taking the drink, and to fast for 3 hours after consuming the drink. Participants submitted before and after photos documented on a photo food diary application (FoodView app) to verify adherence with the above instructions. Meal starting and ending times were extracted from photo timestamps and verified using participant logs in a paper diary. Postprandial glycemic responses (PPG) to the standardized breakfasts were calculated from CGM data as the incremental area under the curve during the 3-hour post-meal period (iAUC_0-3h_). Fasting glucose values were taken as the recorded glucose value immediately before the meal start time. Meals were excluded from the calculation if the total meal duration exceeded 1 hour, or if participants consumed additional food, drink, or caffeine during the 3 hours after the start of the meal.

This procedure was undertaken to simulate postprandial glucose testing in the morning which is standard for OGTT, but on additional days and without subjecting participants to additional blood draws. Accordingly, postprandial glucose was examined in relation to breakfasts only to ensure a sufficient fasting period beforehand. Lunch, dinner, and snacks were not restricted.

### OGTT Procedure (study 2 only)

**Figure 2.**
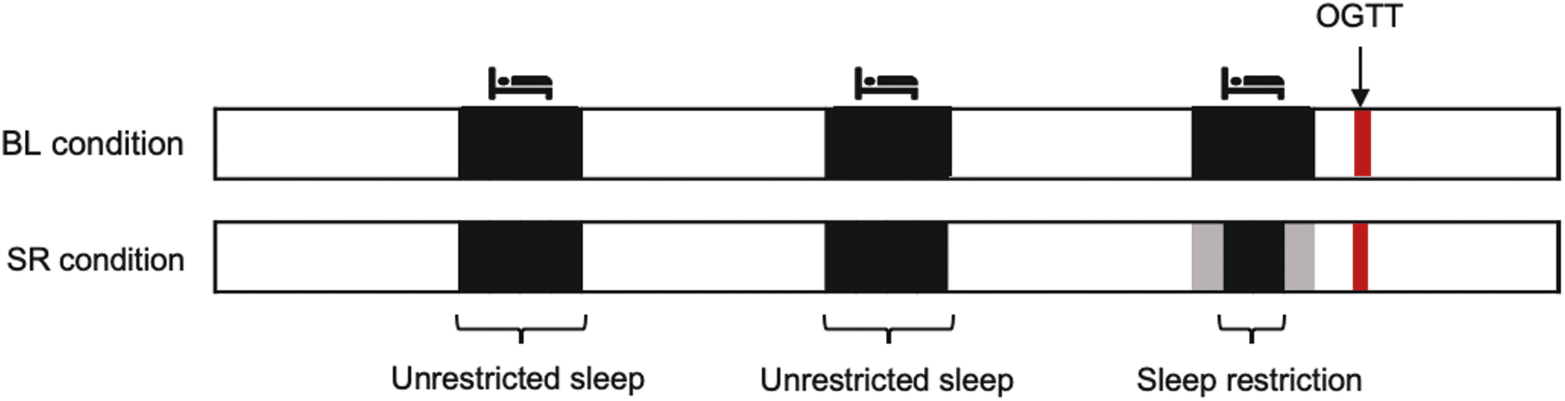
Brief 3-day sleep protocols in control/baseline (BL) condition and sleep restricted (SR) condition used in study 2. OGTTs took place on the morning following the third night of each condition.

Following the free-living study, 56 participants in study 2 additionally completed a follow-up procedure, undergoing one 2-hour OGTT after three nights of ad-libitum sleep (control/baseline [BL] condition), and a second 2-hour OGTT following two nights of ad-libitum sleep and a one night of reduced TIB by 1–2 hours on the night before (sleep restricted [SR] condition; protocol shown in Figure 2). Both OGTTs were separated by a minimum of 1 week.

Participants’ bedtimes and wake times on the three nights before each OGTT were prescribed by the research staff. In the control condition, participants kept to a minimum TIB matching their mean sleep duration in the preceding free-living study on all three nights before the OGTT. In the SR condition, participants were kept to the same minimum TIB on the first two nights to wash out any influences of prior sleep restriction. Bedtimes and wake times on the third night (the night before the OGTT) were adjusted to decrease TIB (relative to the participant’s mean sleep duration in the free-living study) by 1.5–2 hours. This decrease aimed to simulate a situation of a single night of moderate sleep restriction.

Participants wore an OURA ring and self-reported their bedtimes and wake times using a paper sleep diary to verify adherence with the prescribed sleep–wake times on all three nights in each condition. Research staff ascertained participants’ adherence to the sleep schedules prior to the OGTT.

All participants were instructed to fast overnight for at least 8 hours before arriving for the OGTT. On arrival at the laboratory, participants self-reported their level of sleepiness via the Karolinska Sleepiness Scale (KSS). Venous blood was drawn by trained phlebotomists while participants were in a sitting or lying position. Participants were then given a 75-g glucose solution to finish drinking within 5–10 minutes, and blood samples were collected again at 30, 60, 90, and 120 minutes after finishing the solution. An assay of glucose and insulin concentrations was performed by a blood work laboratory.

### Calculating indices of glucose tolerance and insulin sensitivity

Data from one participant whose insulin level exceeded 300 mU/L were excluded from calculations as the exact value could not be determined. Glucose and insulin areas under curves (AUCs) were calculated using the trapezoid rule to measure glucose and insulin excursions in response to the glucose solution challenge during the OGTT [44]. HOMA-IR and Matsuda indices of insulin resistance were calculated using the following formulas, where higher HOMA-IR scores denoted greater insulin resistance [45], and lower Matsuda index scores denoted poorer insulin sensitivity [46].


$$\begin{array}{l} HOMAIR=\frac{Fasting\;plasma\;glucose\times Fasting\;plasma\;insulin}{22.5} \end{array}$$

$$\begin{array}{l} MI=\frac{10000}{\begin{array}{c} \surd [(Fasting\;plasma\;glucose\times Fasting\;plasma\;insulin)\;\times (mean\;glucose\times mean\;plasma\;insulin)] \end{array}} \end{array}$$

## Statistical Analyses

### Data preparation

CGM data from three participants in study 1 were excluded due to faulty sensors, leaving a final sample of 116 participants examined in the following analyses.

Sleep–wake timings were extracted from Oura’s cloud API and manually reviewed using a semi-automated process that flagged and adjusted sleep onset or offset times that were inconsistent with participants’ self-reported sleep timings. (A section of the code enabling these functions has been provided in an earlier publication [42]). In study 1, a total of 847 nights had a qualifying sleep period (average per participant = 11.8 ± 1.7 nights; percentage missing = 10%) while CGM data were collected on 876 days (average per participant = 12.2 ± 2.0 nights; percentage missing = 6%). Both sleep and CGM data were available on 804 days (average per participant = 11.2 ± 2.3 nights; percentage missing = 14%). In study 2, a total of 714 nights had a qualifying sleep period (average per participant = 12.3 ± 1.0 nights; percentage missing = 5%) while standardized breakfast PPG was available on 368 days (average per participant = 6.3 ± 1.3 nights; percentage missing = 14%). Both sleep and PPG data were available on 350 days (average per participant = 6.0 ± 1.5 nights; percentage missing = 14%).

Group-mean centering was performed on all daily nocturnal sleep variables by subtracting the participant’s mean from the daily value of a given sleep measure, decomposing each variable into a level 2 predictor representing between-person effects (the participant mean) and level 1 predictor representing within-person effects (deviation from that participant’ mean). The level 2 variables were centered to the grand mean by subtracting the mean value of that parameter across all nights.

### Predicting same-night–next-day glucose characteristics under standardized dietary conditions

The following analyses were performed using the “lme4” (ver1.1-27.1) and “lmerTest” (ver3.1-3) packages implemented using R version 4.0.2. Data were examined using linear mixed-effects modeling approach to account for heterogeneity between participants in glucose characteristics. Maximum likelihood estimation was used to estimate variance components.

Using data collected from participants in study 1, separate models were constructed to examine the effects of TST, MST, and SE on next-day means and SD of glucose measured across each 24-hour period (0800–0800 hours), nocturnal period (2400–0800 hours; same night), and daytime period (0800–2400 hours). For comparison, an identical analysis of glucose during the nocturnal period was performed using data from participants in study 2 who were not restricted in what they ate for dinner or for night snacks. Square-root transformations were performed where needed to meet assumptions of normally distributed residuals. Models were adjusted for age, sex, BMI, and self-reported nap duration on the previous day. To account for possible temporal trends in glucose values resulting from instrumentation drift, an ordinal variable accounting for the day of wearing the CGM (CGMday) was included as a covariate to anchor all effects to the first day of measurement. Higher-order polynomial terms for CGMday were added where significant to account for possible non-linear trends in glucose data over time. *p*-values were corrected for multiple comparisons using the Benjamini-Hochberg procedure [47].

Cohen’s f^2^ effect sizes were calculated for each independent within-participant sleep predictor using the formula f^2^ = (R_AB_^2^ -R_A_^2^)/(1-R_AB_^2^), where B denotes the sleep predictor of interest, A denotes other predictor variables; R_AB_^2^ denotes the proportion of variance accounted for by both A and B, and R_A_^2^, obtained by dropping the relevant sleep predictor from the model, denotes the proportion of variance accounted for by A [48]. Effect sizes were interpreted using the guideline: small: f^2^ ≥ 0.02, medium: f^2^ ≥ 0.15, large: f^2^ ≥ 0.35 [49].

### Predicting postprandial glycemic responses to standardized breakfasts

Using data collected from participants in study 2, separate models examined the effects of TST, MST, and SE on next-morning PPG to standardized breakfasts (iAUC_0-3h_). Models were adjusted for age, sex, BMI, and self-reported nap duration on the previous day.

### Linear regression models

For the eleven participants who participated in both study 1 and 2, data from both studies were combined into a single record. Aggregated multiple linear regression models examined the effects of intraindividual sleep variability (TST SD) on mean and SD of glucose measured across the 24-hour period, nocturnal period, and daytime period among participants in study 1, and on PPG to standardized breakfasts among participants in study 2 averaged across all days. All models were adjusted for mean TST, age, sex, and BMI.

### OGTT outcomes following baseline and restricted sleep conditions

Repeated measures analyses of variance (ANOVAs) were performed using SPSS Statistics (Ver 27.0.1.0, SPSS, Chicago, IL) to compare insulin resistance indices HOMA-IR and Matsuda index, glucose AUC and insulin AUC following the control and SR conditions. Two-way repeated measures ANOVAs were used to compare glucose and insulin concentrations across time (0, 30, 60, 90, and 120 minutes) between control and SR conditions.

## Results

### Participant demographics

Participants were of comparable age, BMI, and sex ratio across both studies. Mean glucose across the whole day, nocturnal period daytime period, and SD of nocturnal glucose were higher in study 2 than in study 1 (all *p*s < .05; Table 1; Supplementary Figure 2). The consistently higher average glucose levels across the day in study 2 (approximately 10 mg/dL) likely reflect differences in CGM instrumentation between study 1 and 2.

**Table 1.** Characteristics of Participants Recruited Into Study 1 and 2

	Study 1 (*n* = 72)^1^	Study 2 (*n* = 47; Study 2 alone)
Female	39 (54.2%)	25 (53.2%)
Age (years)	22.16 (1.74)	23.12 (1.59)
BMI (kg/m^2^)	20.94 (2.35)	21.35 (2.19)
Parental history of diabetes	3 (4.2%)	4 (8.5%)
*Glucose characteristics (measured by CGM)*		
Mean over 24 h (mg/dL)	85.70 (7.57)^*^	98.49 (7.00)^*^
SD over 24 h (mg/dL)	18.70 (3.86)	18.06 (3.36)
Nocturnal glucose mean (mg/dL)	71.94 (7.99)^*^	87.89 (7.18)^*^
Nocturnal glucose SD (mg/dL)	6.64 (2.44)^*^	9.36 (2.55)^*^
Daytime glucose mean (mg/dL)	92.14 (7.78)^*^	103.46 (7.55)^*^
Daytime glucose SD (mg/dL)	18.85 (4.44)	18.50 (3.63)
Fasting glucose (mmol/L)	4.08 (0.49)	4.69 (0.37)
*Glucose characteristics (measured by venous blood)*		
HbA1c (%)	5.22 (0.26)	—
Fasting glucose (mmol/L)	4.64 (0.34)	4.49 (0.41)
2-h postprandial glucose (mmol/L)	5.16 (1.24)	4.95 (1.27)
Fasting insulin (mU/L)	—	5.55 (2.41)
2-h postprandial insulin (mU/L)	—	43.08 (35.04)
*Sleep characteristics (measured by OURA ring)*		
Average bedtime	1:33 ± 1:04	1:42 ± 1:05
Average wake time	8:22 ± 0:42^*^	9:02 ± 1:08^*^
Average mid-sleep time (MST)	4:58 ± 0:48	5:22 ± 1:04
Average time in Bed (h)	6.82 ± 0.82^*^	7.35 ± 0.67^*^
Average total sleep time (h)	5.83 ± 0.83^*^	6.17 ± 0.70^*^
Average sleep efficiency (%)	85.62 ± 5.24	84.05 ± 4.08
Average sleep onset latency (min)	10.09 ± 3.40	10.05 ± 3.07
Average wake after sleep onset (min)	40.39 ± 20.51^*^	50.59 ± 17.71^*^
Total sleep time *SD* (min)	52.3 ± 19.6	60.6 ± 24.2
Bedtime *SD* (min)	56.1 ± 30.5	58.5 ± 26.5
Wake time *SD* (min)	36.1 ± 25.7	70.1 ± 36.9
Mid-sleep time *SD* (min)	38.8 ± 24.1	54.2 ± 26.3

BMI, body mass index; CGM, continuous glucose monitor; *SD*, standard deviation.

^1^Data from 11 participants who participated in both Study 1 and 2 were included in this tabulation of data from Study 1.

^*^ *p*-value < .05.

### Associations between sleep and glucose profiles under standardized dietary conditions (study 1)

Among 69 individuals examined from Study 1, means and SDs of glucose values during the daytime period or across 24 hours were not associated with any sleep variables (all *p*s > .05; Table 2). Sleep variables also did not affect mean nocturnal glucose levels at either the within or between-participant levels. However, longer sleep durations and earlier sleep times were associated with lower nocturnal glucose variability both at the between and within-participants levels (all *p*s < .001, Table 2). While these latter associations survived multiple comparison corrections, the predicted deviations in glucose levels were only <1mg/dL for each hourly change in TST and midsleep time. As such, this finding may not be functionally relevant.

**Table 2.** Associations Between Sleep and Glucose Characteristics in Study 1 (Controlling for Age, Sex, BMI, Day of Study, Previous Day Nap Duration)

		Across 24 hours		Nocturnal glucose (12 am–8 am)		Daytime glucose (8 am–12 am)	
		Mean^b^ β ± SE (*P*-value) [Cohen’s f^2^]	*SD* ^a^ β ± SE (*P*-value) [Cohen’s f^2^]	Mean β ± SE (*P*-value) [Cohen’s f^2^]	*SD* ^a^ β ± SE (*P*-value) [Cohen’s f^2^]	Mean β ± SE (*P*-value) [Cohen’s f^2^]	*SD* ^a^ β ± SE (*P*-value) [Cohen’s f^2^]
TST	Between	0.09 ± 0.10 (.381)	−0.03 ± 0.06 (.672)	−1.00 ± 1.01 (.327)	**−0.21 ± 0.06** (<.001)^*^	−0.78 ± 0.99 (.434)	−0.02 ± 0.07 (.827)
	Within	−0.01 ± 0.03 (.710) [0.00]	−0.01 ± 0.02 (.462) [0.00]	−0.12 ± 0.28 (.678) [0.00]	**−0.16 ± 0.02** (<.001)^*^ [0.07]	0.06 ± 0.28 (.819) [0.00]	−0.00 ± 0.02 (.852) [0.00]
MST	Between	−0.22 ± 0.12 (.075)	0.03 ± 0.07 (.627)	3.14 ± 1.15 (.008)	**0.31 ± 0.07** (<.001)^*^	1.77 ± 1.16 (.133)	0.06 ± 0.08 (.454)
	Within	−0.00 ± 0.03 (.966) [0.00]	−0.03 ± 0.02 (.204) [0.00]	0.71 ± 0.32 (.028) [0.00]	**0.14 ± 0.03** (<.001)^*^ [0.04]	−0.21 ± 0.32 (.517) [0.00]	−0.03 ± 0.02 (.273) [0.00]
SE	Between	0.00 ± 0.02 (.902)	0.01 ± 0.01 (.301)	−0.10 ± 0.17 (.554)	−0.00 ± 0.01 (.685)	0.04 ± 0.16 (.804)	0.01 ± 0.01 (.343)
	Within	−0.00 ± 0.00 (.451) [0.00]	0.00 ± 0.00 (.287) [0.00]	−0.00 ± 0.06 (.937) [0.00]	−0.00 ± 0.01 (.709) [0.00]	0.02 ± 0.06 (.666) [0.00]	0.01 ± 0.00 (.221) [0.00]

TST, total sleep time; MST, mid-sleep time; SE, sleep efficiency.

^a^Dependent variables were square-root transformed using the formula √ x.

^b^Dependent variables were square-root transformed using the formula √ (max(x)—x).

^*^Adjusted *p*-value < .05 using the Benjamini-Hochberg method (bold).

No significant associations were found at the between-participant level. In all models, self-reported nap durations were not associated with same-night or next-day glucose characteristics (all *p*s > .05).

### Associations between sleep and glucose profiles in study 2

There were no significant associations when examining how sleep variables were associated with PPGs to standardized breakfasts among 58 participants in study 2 at the between-participant or within-participant level (all *p*s > .05). In all models, self-reported nap durations were not associated with same-night or next-day glucose characteristics (all *p*s > .05). As in Study 1, a statistically significant but functionally irrelevant association between longer sleep duration and lower nocturnal glucose variability was found (*p* < .001; Table 3).

**Table 3.** Associations Between Sleep and Nocturnal Glucose Characteristics in Study 2 (Controlling for Age, Sex, BMI, Day of Study, Previous Day Nap Duration)

		Mean β ± SE (*P*-value) [Cohen’s f^2^]	SD^a^ β ± SE (*P*-value) [Cohen’s f^2^]
TST	Between	−2.38 ± 1.31 (.075)	−0.15 ± 0.07 (.041)
	Within	−0.61 ± 0.29 (.035) [0.01]	**−0.12 ± 0.03** (<.001)^*^ [0.03]
MST	Between	1.60 ± 0.88 (.077)	**0.17 ± 0.04** (<.001)^*^
	Within	0.79 ± 0.31 (.011) [0.01]	0.08 ± 0.03 (.010) [0.01]
SE	Between	−0.26 ± 0.22 (.224)	−0.00 ± 0.01 (.813)
	Within	−0.02 ± 0.06 (.747) [0.00]	0.00 ± 0.01 (.999) [0.00]

TST, total sleep time; MST, mid-sleep time; SE, sleep efficiency.

^a^Dependent variables were square-root transformed using the formula √ x.

^*^Adjusted *p*-value < .05 using the Benjamini–Hochberg method (bold).

### Associations between sleep variability and CGM outcomes

Greater sleep variability, correcting for average sleep duration was associated with higher average nocturnal glucose variability (*β* = 1.94, *p* = .02). However, this association was not significant after correcting for multiple comparisons. Sleep variability was not significantly associated with daytime or whole-day glucose profiles measured in study 1 or with PPGs to standardized breakfasts in study 2 (all *p*s > .05; Supplementary Table 1).

### Effects of moderate sleep restriction on OGTT (*n* = 56)

**Figure 3.**
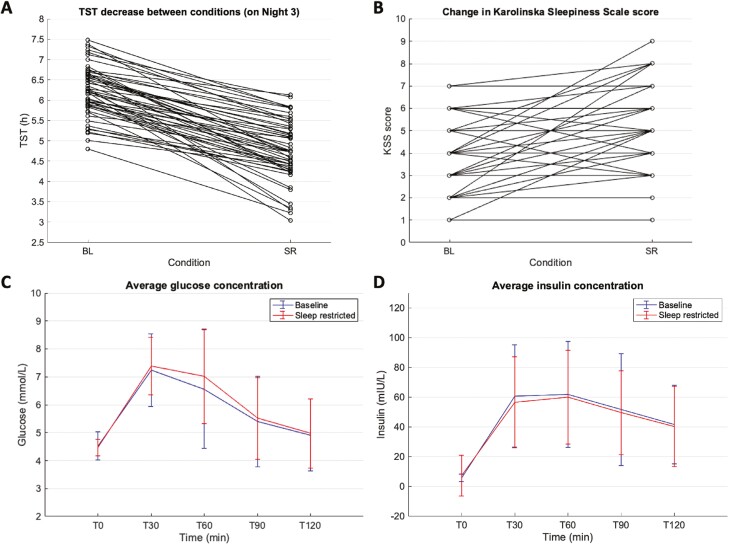
Effects of moderate sleep restriction on oral glucose tolerance test (OGTT; *n* = 56) (A–B) total sleep time (TST) change on the night before OGTT and Karolinska Sleepiness Scale (KSS) score change on the morning of OGTT between baseline (BL) and sleep restricted (SR) conditions. (C–D) Glucose and insulin concentrations across time points (30-minute intervals) in the 2-hour OGTT.

Relative to sleep durations in the ad libitum sleep condition (TIB Mean ± *SD* = 7.48 ± 0.88; TST Mean ± *SD* = 6.22 ± 0.62 hours), participants successfully reduced TIB by an average of 1.73 hours (*SD* = 0.97) and TST by an average of 1.45 hours (*SD* = 0.68) on the night before the OGTT in the sleep-restricted condition (*p* < .001; Figure 3). Participants also reported being sleepier in the sleep-restricted condition (Mean KSS ± *SD* = 4.89 ± 1.91) compared to the ad libitum sleep condition (Mean KSS ± *SD* = 3.64 ± 1.49).

HOMA-IR and Matsuda indices of insulin sensitivity did not differ between control and sleep-restricted conditions (F (1,54) = 0.38, *p* = .54; F (1,54) = 0.41, *p* = .52, respectively). Glucose and insulin AUCs were not statistically different between conditions (glucose: F (1,54) = 3.07, *p* = .09; insulin: F (1,54) = 0.08, *p* = .77). Glucose and insulin concentrations over time were also not statistically different between conditions (glucose: F (1,54) = 2.74, *p* = .10; insulin: F (1,54) = 0.03, *p* = .87), nor were significant interactions found between OGTT timepoints and condition (glucose: F (4,216) = 2.48, *p* = .06; insulin: F (4,216) = 0.62, *p* = .58; Figure 3).

## Discussion

We studied a sample of healthy university students who underwent up to 2 weeks of sleep and CGM in free-living conditions. There was no significant effect of nocturnal sleep on following-day blood glucose profiles under standardized dietary conditions, or on postprandial glucose excursions following a standardized breakfast in unrestricted dietary conditions. These findings held when naps were taken into consideration. Even when sleep was deliberately restricted, glucose excursions in response to a glucose load were neither significantly elevated compared to baseline, nor was the maintenance of blood sugar levels accompanied by higher than baseline insulin levels. Our findings were unlikely to arise from insufficient variation in sleep duration as there were previously observed effects on mood, motivation, and sleepiness [42]. Taken together, these results suggest that glucose metabolism in healthy college students may be more resilient to real-life fluctuations in sleep duration than previously thought.

Existing work collectively suggests that reduced sleep duration or quality of sleep has negative impacts on glucose metabolic health and risk of diabetes [1–6]. In many lab-based investigations, partial sleep deprivation is often associated with altered metabolic outcomes linked with poorer glucose regulation or energy metabolism. These include decreased glucose tolerance [9], lowered insulin sensitivity [7, 8], elevated cortisol levels [9], hormonal imbalance [50], and systemic inflammation [8, 51, 52]. Evidence from these experimental studies supports the narrative that losing sleep is detrimental to one’s metabolic health and may contribute to an increased risk of developing diabetes. However, in artificial lab-based settings, the degree of sleep curtailment is often exaggerated, and very few studies have tested whether these results still hold true when examined in relation to less extreme departures from habitual sleep durations experienced by students or working adults. Our results suggest that the findings of laboratory studies may not apply to relatively healthy college students. Thus, while useful as proof-of-concept illustrations, highly controlled lab studies may be less relevant in real-world settings.

The absence of significant associations between nocturnal sleep and next-day glucose profiles in our study contrasts with research that examined the effects of sleep on CGM-derived glucose profiles in real-world settings, but among individuals with diabetes [20, 23] or older, non-diabetic adults [26]. In persons with type 1 or 2 diabetes, two recent studies showed that nights with longer sleep duration or lower sleep fragmentation were linked with lower glycemic variability the following day [20, 23], while another study in healthy older persons (approximately 25 years older than the present sample) showed that nights with poorer SE and later bedtimes were linked with larger postprandial glycemic responses to breakfast the following morning [26]. As the two studies in diabetic individuals did not control dietary intake, we cannot rule out the possibility that the effects reported previously were contributed by less healthy food choices or higher food intake arising from sleep curtailment. However, in study 2 which had no restriction on nighttime meals, there were still no effects of sleep duration on mean nocturnal glucose levels or postprandial glycemic responses on the following morning. Another possible explanation for our divergent findings is that persons with diabetes and older adults are more adversely affected by short sleep compared to healthy young adults, whose metabolism may be more resilient. For example, insulin secretion and insulin sensitivity of skeletal muscles decline with age and are accompanied by rising glucose levels [53–55]. This motivated us to investigate whether glucose levels were kept in check by higher insulin secretion in healthy younger adults in study 2. However, this turned out not to be an explanation.

Could excursions in real-world sleep durations be insufficient to metabolically tax our Asian sample, whose habitual sleep durations were shorter than Western norms? Of note, only 48% of our participants obtained an average TST of ≥6 hours on weekdays, while 60% obtained this on weekends—well short of the recommended 7-hour minimum per night recommended for individuals of this age group [56]. This is comparable to what has been shown by other Asian samples [57, 58]. Large-scale studies with objective sleep measurement have demonstrated that East Asians sleep significantly less than their counterparts in Europe and Oceania [18, 58–60]. This could lead some to the conclusion that East Asians are genetically more capable of withstanding short sleep. However, epidemiologic data do not bear this expectation out, instead suggesting that the “j” or “u”-shaped relationship between sleep duration and all-cause mortality is similar across the world [61]. Our prior work on adolescents also indicates that when sleep schedules with reduced sleep opportunities are carefully observed, Asian adolescents appear to require the same amount of sleep suggested by Western standards to stay vigilant [62].

More importantly, within the same sample, we previously reported that nights with shorter sleep durations relative to the participant’s average were associated with worse self-reported mood, motivation, and sleepiness the next day [42]. These effects were temporally specific to the day immediately following the night of relatively shorter sleep and did not affect the second day. The sleep schedules reported here were therefore sufficiently taxing to perturb markers of mental well-being.

The contrast in findings between glucose and mental well-being outcomes provides the first, within-sample evidence for differential effects of sleep duration on mental well-being and metabolism. This idea of dissimilar sleep needs for different organ systems has been proposed but has hitherto not been empirically supported with real-world data [63].

Finally, among persons with diabetes, high intra-day glucose variability—which may include hyperglycemic and hypoglycemic excursions [64]—has been linked with an increased risk of heart disease and diabetic complications independent of average glucose levels [65]. However, the functional relevance of changing nocturnal glucose variability in relation to sleep duration and sleep timing here is unlikely, given that excursions were small (each hourly increase in sleep duration was associated with a 0.04 mg/dL decrease in glucose variability).

### Strengths and limitations of the study

Using unobtrusive, objective methods of multi-day sleep tracking and concurrent monitoring of blood glucose levels using CGM, we explored associations between variations in sleep and glucose in a sample of free-living healthy young adults. Although many persons now wear both devices, systematic data collection to inform about lifestyle choices remains surprisingly scant. While nobody should dispute the importance of sleep on health, the results suggest caution when extrapolating to real-life the findings from studies where more extreme levels of sleep restriction were evaluated. Since the majority of experimental studies examined the effects of multiple nights of sleep restriction, it may be that *successive,* as opposed to isolated nights of sleep restriction, e.g. 3 days of TIB restriction by 1–3 hours each night, may be necessary to elicit higher insulin response to a 2-hour OGTT as well as decreased insulin sensitivity measured the next morning [7–9, 66]. While future studies to extend or refute our results await to be performed, it should be borne in mind that even persons who sleep short at night tend to break a run of successive nights of short sleep by interposing nights with longer sleep—resulting in higher weekday sleep variability [18] or by taking afternoon naps [42].

Our consideration of daytime naps in addition to nocturnal sleep in our models is unique among studies on glucose metabolism and sleep. Short mid-afternoon naps have significant neurocognitive benefits, although we did not show any for glucose metabolism here [67].

Another unique feature of this work was the implementation of standardized diets across participants in study 1. As dietary macronutrient composition can contribute significant variance to excursions in glucose levels [68], this circumvents a concern that elevated glucose may be a result of shorter sleep triggering higher energy intake as well a tilt to consuming more flavorful and unhealthy foods [29, 31, 32]. A potential weakness of this design is that the restriction of additional snacks, sugary drinks, and nighttime eating could fail to reflect the true habits of college students.

In view of these limitations, our findings should not be taken as a license for college students to indulge in shortened and irregular sleep. As we selected participants who were generally lean, healthy, and had normal glucose tolerance, to begin with, these results should also not be extrapolated to less healthy young adults or older adults.

## Conclusion

We showed that in East Asian, healthy college students, realistic fluctuations in day-to-day sleep duration, even after factoring in naps, were not associated with adverse effects on glucose levels. An added night of moderate sleep restriction was also not found to affect insulin levels or insulin sensitivity. These highlight the importance of expanding investigations beyond the laboratory into realistic field settings to help us better understand the practical implications of common sleep habits on glucose handling in non-diabetic individuals.

## Supplementary material

Supplementary material is available at *SLEEP* online.

zsae224_suppl_Supplementary_Figures_1-2_Tables_1

## Data Availability

The data in this article are available upon reasonable request from the authors.
