# Supplementary material for: Effects of night-to-night variations in objectively measured sleep on blood glucose in healthy university students
Source: Sleep. 2024 Sep 26;48(2):zsae224. doi: 10.1093/sleep/zsae224 (PMC11807882; doi:10.1093/sleep/zsae224)
Supplement: zsae224_suppl_Supplementary_Figures_1-2_Tables_1 [file zsae224_suppl_supplementary_figures_1-2_tables_1.docx]

**SUPPLEMENTARY MATERIALS**

**Effects of night-to-night variations in objectively measured sleep on blood glucose in healthy university students**

Alyssa S. C. Ng^1^, E Shyong Tai^2^, Michael W. L. Chee^1^

^1^Sleep and Cognition Laboratory, Centre for Sleep and Cognition, Yong Loo Lin School of Medicine, National University of Singapore, Singapore

^2^ Department of Medicine, Yong Loo Lin School of Medicine, National University of Singapore, Singapore

**Corresponding Author Information**

Prof Michael W.L. Chee
Sleep and Cognition Laboratory, Centre for Sleep and Cognition, Yong Loo Lin School of medicine, National University of Singapore
Tahir Foundation Building, MD1
12 Science Drive 2, Singapore 117549
Email: michael.chee@nus.edu.sg

Compliance to standardized meals

Seventy-two participants in Study 1 provided photo logs of the standardized breakfasts, lunches and dinners on a total of 1008 days (14 days per person). Of these, 899 breakfasts (89%), 918 (91%) lunches and 906 (90%) dinners were determined by research staff to be compliant (following the criteria listed under methods; see Suppl Fig 1 for visual aid). Overall, a total of 812 out of 1008 days were found to have no non-compliant meals (mean per person = 11.3 days).

Fifty-eight participants in Study 2 provided photo logs of a total of 383 standardized breakfasts (mean per person = 6.6). Fifty-nine breakfasts were excluded when calculating the breakfast PPG due or consumption of additional food, drink or caffeine within 3 hours of consuming the breakfast, leaving a remainder of 347 breakfast PPGs included in the final analyses.


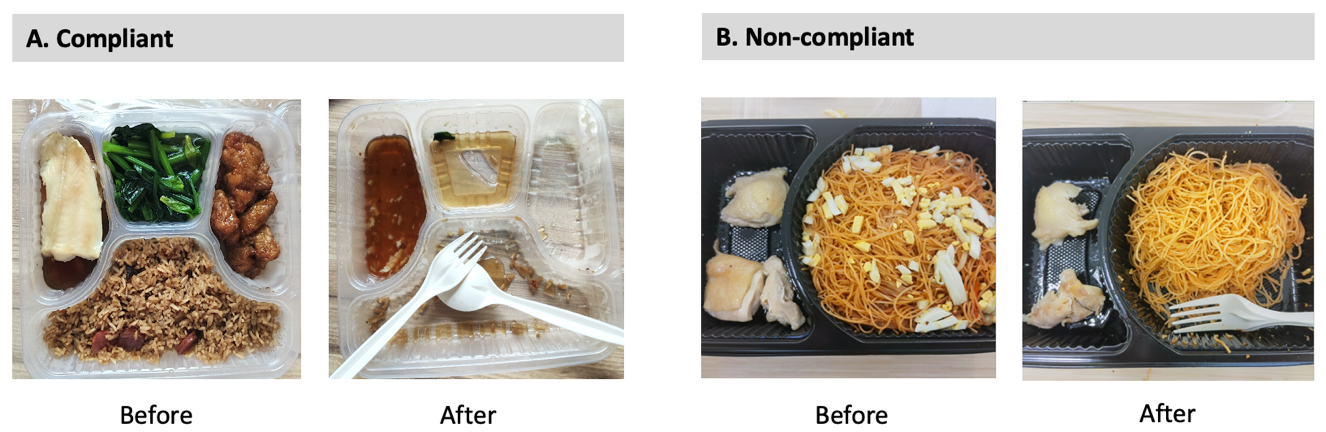


**Suppl Fig 1.** Example of a compliant meal (A) and non-compliant meal (B) in Study 1.

**
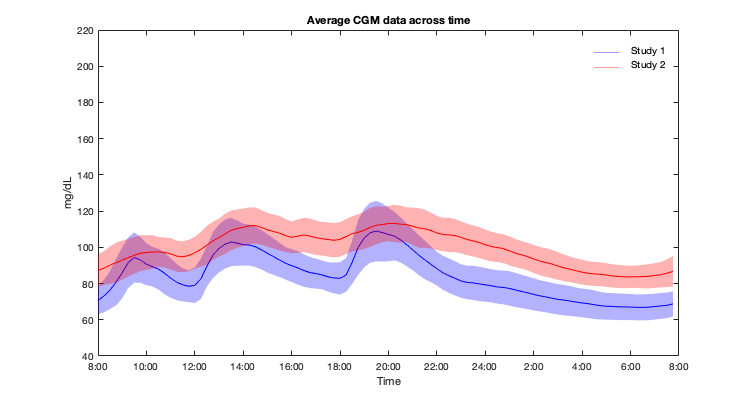
Suppl. Fig 2.** Glucose levels across time averaged across participants in Study 1 and Study 2. Error bands represent one standard deviation of mean values. Pronounced peaks in glucose levels corresponding to the catered breakfast, lunch and dinner windows can be clearly seen in Study 1, while the flatter distribution of glucose levels in Study 2 suggests that students food intake times were more variable across the 24-hour day when there was no restriction. Higher overall glucose levels in Study 2 may be attributed to the use of different CGM instruments between studies.

| **Suppl. Table 1.** Associations between mean TST and TST variability and mean CGM outcomes in Study 1, mean PPGR in Study 2 (adjusted for age, sex, BMI) | | | | | | |  |
| --- | --- | --- | --- | --- | --- | --- | --- |
|  | Across 24h | | Nocturnal glucose  (12AM-8AM) | | Daytime glucose  (8AM-12AM) | |  |
|  | Mean  β ± SE  (P-value)  [Cohen’s f^2^] | SD  β ± SE  (P-value)  [Cohen’s f^2^] | Mean  β ± SE  (P-value)  [Cohen’s f^2^] | SD  β ± SE  (P-value)  [Cohen’s f^2^] | Mean  β ± SE  (P-value)  [Cohen’s f^2^] | SD  β ± SE  (P-value)  [Cohen’s f^2^] | Breakfast PPGR  β ± SE  (P-value)  [Cohen’s f^2^] |
| TST SD (h) | 0.10±2.88  (0.974)  [0.00] | 0.13±1.39  (0.924)  [0.00] | 1.22±3.00  (0.685)  [0.00] | 1.94±0.84  (0.024)  [0.05] | -0.71±2.95  (0.810)  [0.01] | 0.48±1.64  (0.771)  (0.03) | 3.05±15.29  (0.843)  [0.00] |
| TST mean (h) | -0.72±1.24  (0.562)  [0.00] | -0.02±0.60  (0.977)  [0.00] | -0.92±1.29  (0.478)  [0.01] | -0.88±0.36  (0.017)  [0.09] | -0.76±1.27  (0.553)  [0.01] | 0.27±0.71  (0.706)  [0.00] | -7.18±8.46  (0.400)  [0.01] |
| *Adjusted p-value < 0.05 using the Benjamini Hochberg method | | | | | | |  |
